# Comparative Analysis of the Transcriptome and Distribution of Putative SNPs in Two Rainbow Trout (*Oncorhynchus mykiss*) Breeding Strains by Using Next-Generation Sequencing

**DOI:** 10.3390/genes11080841

**Published:** 2020-07-24

**Authors:** Lidia de los Ríos-Pérez, Ronald Marco Brunner, Frieder Hadlich, Alexander Rebl, Carsten Kühn, Dörte Wittenburg, Tom Goldammer, Marieke Verleih

**Affiliations:** 1 Institute of Genetics and Biometry, Leibniz Institute for Farm Animal Biology (FBN), Wilhelm-Stahl-Allee 2, 18196 Dummerstorf, Germany; perez@fbn-dummerstorf.de (L.d.l.R.-P.); wittenburg@fbn-dummerstorf.de (D.W.); 2 Institute of Genome Biology, Leibniz Institute for Farm Animal Biology (FBN), Wilhelm-Stahl-Allee 2, 18196 Dummerstorf, Germany; brunner@fbn-dummerstorf.de (R.M.B.); hadlich@fbn-dummerstorf.de (F.H.); rebl@fbn-dummerstorf.de (A.R.); tom.goldammer@uni-rostock.de (T.G.); 3 Institute of Fisheries, Mecklenburg-Vorpommern Research Centre for Agriculture and Fisheries (LFA MV), 18069 Rostock, Germany; c.kuehn@lfa.mvnet.de; 4 Faculty of Agriculture and Environmental Sciences, University of Rostock, 18059 Rostock, Germany

**Keywords:** rainbow trout, transcriptome, RNA-seq, single nucleotide polymorphism, SNP, p53, aquaculture, selective breeding

## Abstract

Selective breeding can significantly improve the establishment of sustainable and profitable aquaculture fish farming. For rainbow trout (*Oncorhynchus mykiss*), one of the main aquaculture coldwater species in Europe, a variety of selected hatchery strains are commercially available. In this study, we investigated the genetic variation between the local Born strain, selected for survival, and the commercially available Silver Steelhead strain, selected for growth. We sequenced the transcriptome of six tissues (gills, head kidney, heart, liver, spleen, and white muscle) from eight healthy individuals per strain, using RNA-seq technology to identify strain-specific gene-expression patterns and single nucleotide polymorphisms (SNPs). In total, 1760 annotated genes were differentially expressed across all tissues. Pathway analysis assigned them to different gene networks. We also identified a set of SNPs, which are heterozygous for one of the two breeding strains: 1229 of which represent polymorphisms over all tissues and individuals. Our data indicate a strong genetic differentiation between Born and Silver Steelhead trout, despite the relatively short time of evolutionary separation of the two breeding strains. The results most likely reflect their specifically adapted genotypes and might contribute to the understanding of differences regarding their robustness toward high stress and pathogenic challenge described in former studies.

## 1. Introduction

Rainbow trout (*Oncorhynchus mykiss*) is one of the most widely used finfish species reared in aquaculture facilities around the world: traditionally in Europe and currently particularly in Chile, Turkey, and Iran [1,2]. It is believed that nearly all cultured strains originated from a wild strain stock from the McLeod River, California [3,4]. As a result of the advanced domestication of this salmonid fish and its selective breeding, a variety of hatchery strains are commercially available. The selection focuses mainly on the economically relevant traits such as growth and survival that ensure breeding with maximized efficiency and minimized losses [5]. Examples of widely used breeding strains are the ones provided by Troutlodge (Tacoma, WA, USA), including the Silver Steelhead that is selected for good growth in both sea-water and fresh-water, or the German Hofer strain (Oberndorf, Germany), which is highly resistant to the whirling disease. 

The selective breeding of fish that are highly robust under difficult environmental conditions can produce offspring with improved and desirable characteristics. The adapted phenotype results from an interplay of environmental stimuli, the individual genotype, and epigenetic modulations such as DNA methylations [6]. Specific environmental conditions determine the epigenetic marks and thus the transcriptional regulation of target genes and influence the phenotype [7,8]. In addition, DNA modifications such as single nucleotide polymorphisms (SNPs) or copy number variations can lead to modified methylation sites and hence to an altered phenotype. One of these adapted breeding strains is the German Born trout. It has its origin in a breeding program that started more than 40 years ago at the Fisheries Institute in Born, Germany [9]. The Born strain was bred out of a Silver Steelhead strain in the brackish water of the Baltic Sea with its abiotic challenges including a characteristic germ spectrum, a defined salinity, and distinct temperature amplitudes [10]. It is highly probable that these environmental stimuli together with individual genotypes have modified, among others, the epigenetic methylation of particular genes in the Born trout strain, which in turn is reflected by altered gene-expression profiles and thus a specific phenotype since the strain appears to be less susceptible to certain stressors than others. In previous transcriptomic studies, we recorded the tissue-dependent expression profiles of the Born trout and identified characteristic signatures that distinguish this strain from the imported Silver Steelheads, used mainly in German aquaculture [10,11,12,13,14,15]. With regard to immune-defense mechanisms, we detected higher levels of innate-immune transcripts encoding, for instance, antimicrobial peptides [10] or particular complement components [16] in the liver of Born trout compared to Silver Steelheads, while the levels of most immune-relevant transcripts were reduced in the spleen of the Born trout [12,16]. After peritoneal infection with the pathogen *Aeromonas salmonicida*, ssp. *Salmonicida*, we identified strain-specific activated pathways in the gills of the Born trout and the Silver Steelheads to cope with the bacterial invasion [15]. Similarly, we identified strain-specific single-gene activations or activated functional pathways for stress caused by high [13,14] or low [11] husbandry temperatures.

The basis for improved breeding is the comprehensive characterization of potential strains such as the Born trout at the molecular genetic level to identify and evaluate the patterns underlying the desirable trait characteristics. In a study from 2012, Gjedrem and colleagues estimated that only 10% of aquaculture production was based on genetically improved breeding stocks and forecast a share of around 50% by 2020 [17]. Despite the enormous potential, the demand and use of genomic tools for breeding purposes seem to not be sufficiently established yet, even for major reared fish species such as rainbow trout [18,19]. Notwithstanding, the knowledge on the genetic background of domesticated rainbow trout hatchery strains greatly improved over the past years, above all also due to the opportunities of next-generation sequencing methods [20,21,22,23]. 

This study aims to investigate the genetic variations between the local rainbow trout selection strain Born, selected for survival, and the commercially available Silver Steelhead Strain, selected for growth by (i) comparing the transcriptome of the liver, muscle, heart, spleen, head kidney, and gill tissue using RNA-seq technologies, and (ii) identifying putative single nucleotide polymorphisms distinguishing both breeding strains. Genetic variations between the two strains can indicate potential specific signatures formed by the anthropogenic selection of the Born strain and serve as models for other strains resulting from similar environmental conditions.

## 2. Materials and Methods

### 2.1. Experimental Animals

The animals used in this study originate from the parallel rearing of two rainbow trout strains at the Institute of Fisheries in Born (LFA MV), Germany. Fish from the imported Silver Steelhead strain (Tacoma, WA, USA) and the local Born strain were grown simultaneously from eggs to 10–11-months-old fish (Steelhead: average length 27.5 ± 2.1 cm, average weight 335.4 ± 66.7 g; Born: average length 26.8 ± 1.4 cm, average weight 280.3 ± 48.8 g). Six tissues (gills, head kidney, heart, liver, spleen, and white muscle) of eight fish of each strain were sampled for further analysis.

### 2.2. Nucleotide Extraction and Library Preparation

Total RNA was extracted from the six tissues of each fish, homogenized in 1 mL TRIzol reagent (Invitrogen, Darmstadt, Germany) and purified using the RNeasy Mini Kit (Quiagen, Hilden, Germany) according to the manufacturer’s protocol. The integrity and quality of the total RNA was evaluated using an Agilent 2100 Bioanalyzer (Agilent Technologies, Waldbronn, Germany) employing the Eukaryote total RNA Nano Series II assay (Agilent Technologies, Santa Clara, CA, USA). Only RNA samples with RNA integrity numbers (RINs) ≥ 8.5 were used for further analysis (Table S1). 

Library constructions followed the TruSeq RNA Sample Preparation v2 Guide supplied with the TruSeq RNA Sample Prep Kit v2 (Illumina, San Diego, CA, USA) with minor modifications. Briefly, 3 µg total RNA was purified to retain only mRNA by using poly-T oligo-attached magnetic beads. Next, mRNA was fragmented and used as a template for cDNA synthesis. The cDNA was subsequently end-repaired and adenylated at the 3′ ends followed by adapter ligation. A PCR approach was used to amplify the amount of DNA in the final library and selectively enrich DNA fragments with successful adapter ligation on both molecule ends. The size and purity of these cDNA libraries were determined using an Agilent 2100 Bioanalyzer. After determining its concentrations on the LightCycler® 96 System (Roche, Basel, Switzerland) using the KAPA SYBR GREEN Library Quantification Kit (Peqlab/VWR brand, Erlangen, Germany), the libraries were diluted to a final DNA concentration of 10 nM. Libraries of each tissue from eight individuals per strain, marked with distinct adapters, were multiplexed and sequenced on Illumina Genome Analyzer GA IIx. Sixty-five cycles paired-end sequencing were conducted using TruSeq SBS Sequencing kits v5 (Illumina). The resulting short sequence reads were transferred to the computer pipeline for further processing.

### 2.3. Sequencing Data Processing and Analysis of Differential Expression

Sequencing reads were trimmed for adapter sequences using the in-house Linux tool Filtrix (unpublished) followed by FastQC [24] quality control checks (≥20 nucleotides, ≥Q30). The resulting high-quality reads were mapped against the reference genome assembly for rainbow trout Omyk_1.0 (GenBank assembly accession GCA_002163495.1, [25]) applying Hisat2 (version 2.1.0, [26]) with the following parameters: paired reads, Softclip, no discordance, and multi maps ≤ 10. The transcript assembly was performed using StringTie [27]. The tool DESeq2 [28] was used to test for differentially expressed (DE) genes. Differential expression between the two rainbow-trout strains was then tested using the Wald’s test of log2 fold changes. Only genes with an absolute fold change (FC) of ≥2.0 (log_2_ FC < −1 or log_2_ FC > 1) and a Benjamini-Hochberg adjusted *p*-value of < 0.05 were deemed differentially expressed. Ingenuity Pathways Analysis (IPA; Ingenuity Systems, Ingenuity, CA, USA) was used to perform functional classifications and enrichment analyses.

### 2.4. Identification of Putative SNPs

We performed FastQC quality control checks of the raw sequencing reads. One sample of gill tissue from Silver Steelhead and one sample of head kidney tissue from Born trout were discarded due to a low number of reads. The identification of putative SNPs was performed following the Genome Analysis Toolkit v4.0 (GATK) [29] pipeline for RNA-seq. Reads were aligned against the rainbow trout reference genome (GCA_002163495.1) using the STAR alignment tool [30]. The obtained SAM files were sorted, and duplicates were marked by Picard tools (). Split’N’Trim, reassign mapping quality, and base recalibration steps were performed according to the GATK pipeline. Variants were called using the HaplotypeCaller tool, which is capable of calling SNPs and Indels simultaneously.

We extracted only the single nucleotide variants and hard-filtered them by excluding those that met the following criteria QualByDepth (QD) < 5.0, FisherStrand (FS) > 30.0, StrandOddsRatio (SOR) > 3.0, RMSMappingQuality (MQ) < 50.0, MappingQualityRankSumTest (MQRankSum) < −10.0, ReadPosRankSumTest (ReadPosRankSum) < −4.0 and clusters of at least three putative SNPs within a window of 35 bases. 

The focus of this study was to find high frequency variants between the two strains. For this purpose, two analyses were performed, analysis 1 (A1) and analysis 2 (A2). A1 aimed to find the variants between the two strains per tissue, and A2 aimed to find the variants between the two strains over all tissues and individuals. For A1, two more filters were applied to the resulting putative SNPs, keeping only those with: (i) call rate of at least 75% per tissue per strain and (ii) each putative SNP had to be homozygous for one of the strains and heterozygous for the other strain, with at least three individuals with the variant. For A2, the applied filters to the resulting putative SNPs were: (i) call rate of at least 90% per strain and (ii) each putative SNP had to be homozygous for one of the strains and heterozygous for the other strain, with at least five individuals with the variant. 

### 2.5. Validation of Putative SNPs by Resequencing 

Putative SNPs distinguishing the Silver Steelhead strain and the Born strain were identified and a set of four was chosen at random for validation out of the homozygous for one of the strains. They could be assigned to exonic regions of the three genes *BTF3* (NC_035081.1, SNP1:15192150, SNP2:15192203), *CIRBP* (NC_035081.1: 58519819), and *FTH1* (NC_035102.1: 22563081). To obtain gene-specific SNP-flanking fragments, we reverse-transcribed 5 μg of RNA of 50 trout from each of the two strains into cDNA using Superscript II (Invitrogen). Samples were randomly selected among those collected over the past 10 years. For each of the 100 samples, an individual tissue mix was used. A standard-PCR reaction was used with the gene-specific oligonucleotide primers listed in Table S2. Sequencing was performed on a MegaBACE capillary sequencer (GE Healthcare). A polymorphic position was defined as validated if the SNP was present in more than one individual and if it was confirmed by the forward and reverse sequence.

### 2.6. Data Deposition

The sequencing data from this study have been submitted to the National Center for Biotechnology Information (NCBI) Sequence Read Archive (SRA) under the BioProject accession number PRJNA638521.

### 2.7. Ethical Statement

The handling and sampling procedures for animals were conducted in compliance with the terms of the German Animal Welfare Act (§ 4(3) TierSchG) and approved by the internal ethics commissions of the Institute of Fisheries, State Research Centre for Agriculture and Fisheries Mecklenburg-Western Pomerania (LFA MV) and the Leibniz Institute for Farm Animal Biology.

## 3. Results and Discussion

### 3.1. A Total of 1760 Annotated Genes Were Differently Expressed Between Rainbow Trout Strains Silver Steelhead and Born

We sequenced RNA samples from six tissues of eight Silver Steelhead and Born rainbow trout each using the Illumina platform. Sequencing data yielded a total of ~346 million paired-end reads, including ~170 million reads for the Silver Steelhead strain and ~175 million reads for the Born strain. The RNA sequencing yielded on average 3.2 million reads per sample. After trimming and quality control, a total of ~324 million high-quality reads (*q* value > 30) were obtained and used to identify DE genes between both strains. The total number of high-quality reads per tissue ranged from ~41 million to ~50 million (Table 1). On average, 87.71% of the high-quality reads aligned to the rainbow trout reference genome (GCA_002163495.1) at unique (73.49%) or multiple positions (14.22%). 

**Figure 1:**
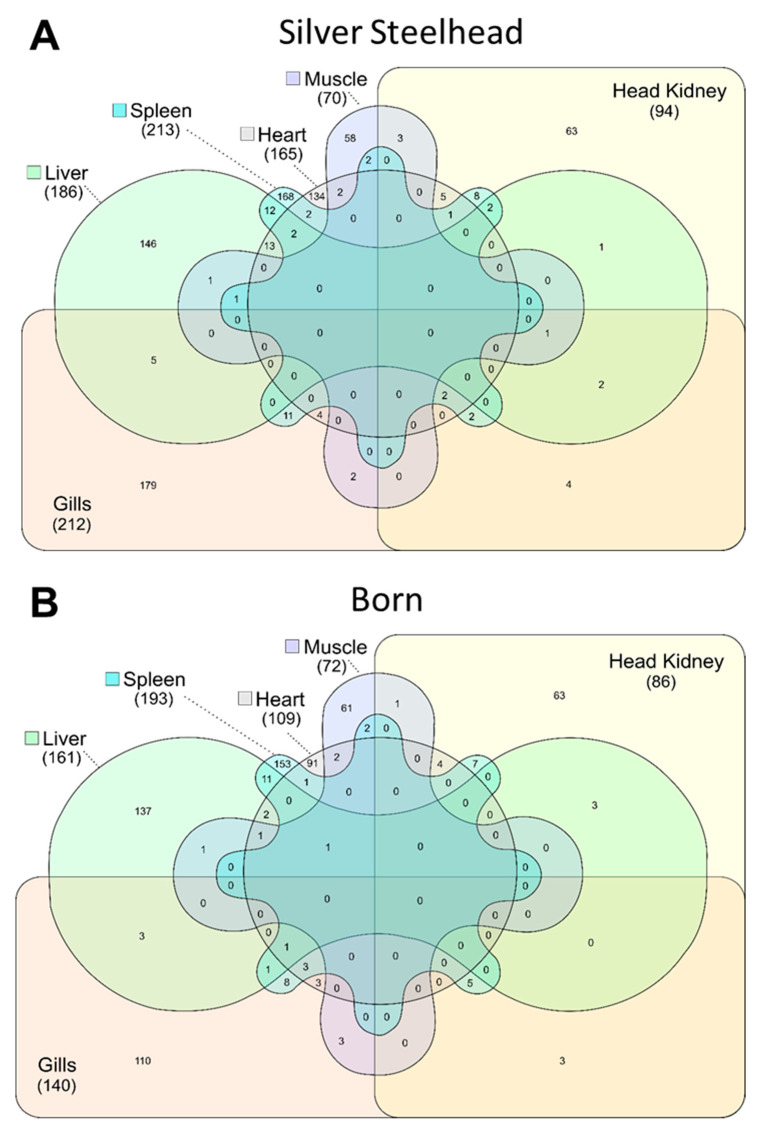
Venn diagram illustrating the common and exclusively expressed genes in the gills, head kidney, heart, liver, spleen, and the white muscle of rainbow trout strain (**A**) Silver Steelhead compared to strain Born, and (**B**) Born compared to strain Silver Steelhead. The total number of differentially expressed genes for each tissue is given in brackets.

We compared the general gene expression of healthy Silver Steelhead and Born trout to detect specific expression patterns that are possibly correlated with the different adaptation potential of both strains. A total of 1760 annotated genes (FC > 2.0, adjusted *p*-value < 0.05) were differentially expressed across all six examined tissues (Table S3). Of these, 976 genes were higher expressed in the Silver Steelhead strain (compared to the Born strain), while 784 genes were higher expressed in the Born strain (compared to the Silver Steelhead strain). This list of DE genes included paralogues/ohnologues genes. For subsequent analysis, we considered only the gene orthologue of the human counterpart. The highest number of genes which were differently expressed in the Silver Steelhead strain compared to the Born strain were detected in the spleen (213), while the lowest number of DE genes were found in the muscle (70). A similar distribution was found for the DE genes in the Born strain compared to the Silver Steelhead strain (193 in the spleen, and 72 in the muscle). The Venn diagrams in Figure 1 depict the tissue-specific and -overlapping features, which were higher expressed in the Silver Steelhead strain compared to the Born strain (Figure 1A) or in the Born strain vice versa (Figure 1B). The total number of DE genes is 9% to 51% higher in the Silver Steelhead strain than in the Born strain in five out of the six tissues. Only in the muscle of the Born strain did we detect 3% more DE genes than in the same tissue of the Silver Steelhead strain.

**Figure 2:**
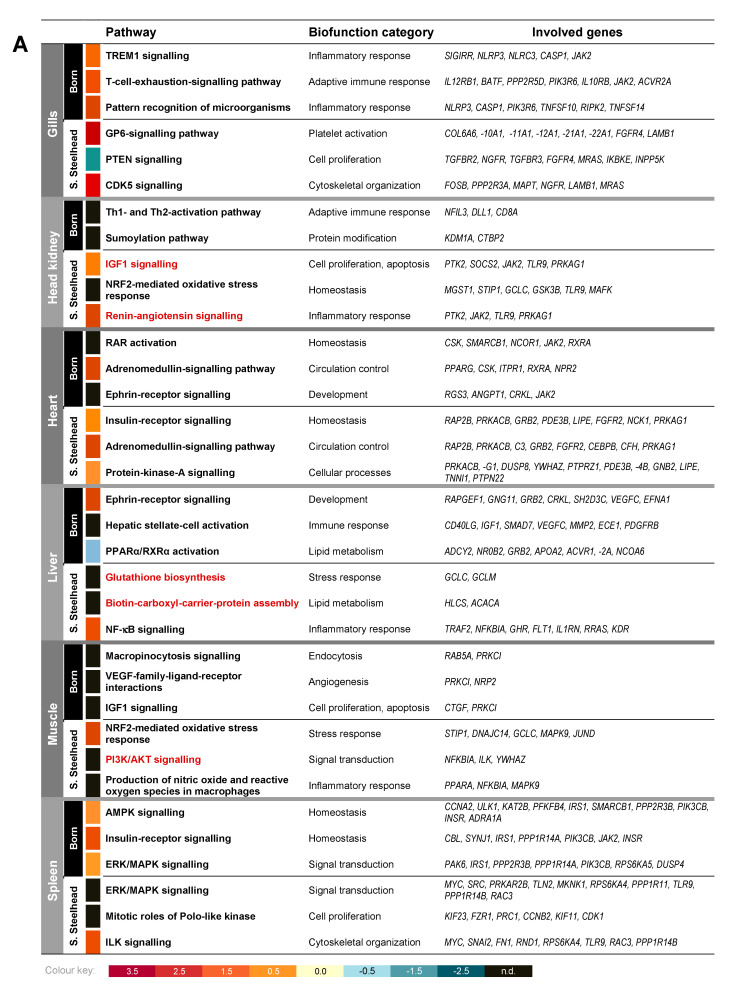

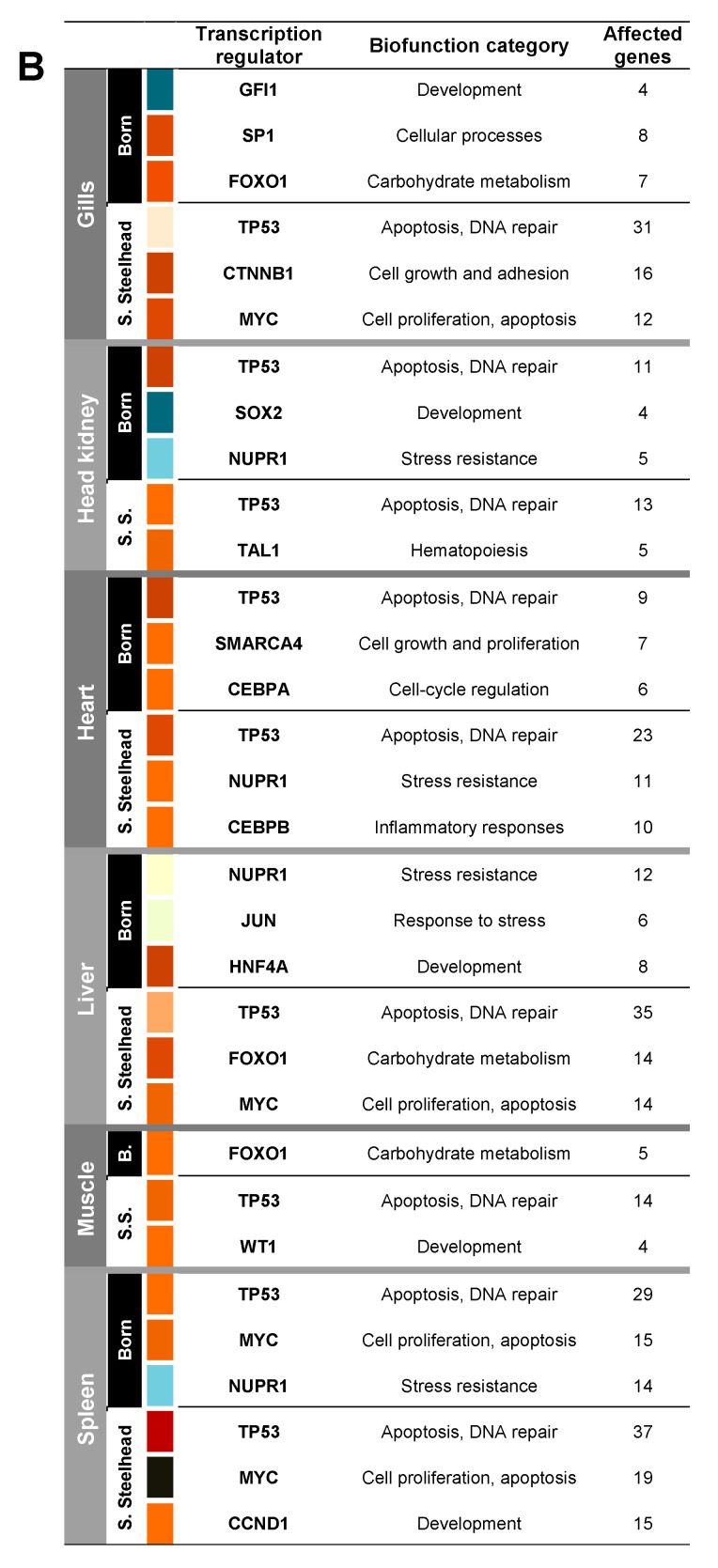
List of the top three significantly enriched strain- and tissue-relevant (**A**) canonical pathways and (**B**) upstream regulators in rainbow trout strains Silver Steelhead and Born. The red font indicates pathways that are unique for one of the strains. The color code below the table A displays the z-score values.

Of the DE genes discriminating both strains, on average about 80% were expressed in a tissue-specific fashion, while the expression of around 20% was detectable in at least two tissues, but at most four tissues. In the Silver Steelhead strain, these tissue-overlapping genes encode the DNA nuclease Harbinger transposase derived 1 (HARBI1) [31] and two proteins involved in the protection against oxidative stress, glutamate cysteine ligase, catalytic subunit (GCLC) [32] and peroxiredoxin 6 protein (PRDX6) [33]. In the Born strain, these tissue-overlapping genes encode NLR family CARD domain containing protein 3 (NLRC3), a central negative regulator of the inflammatory immune response [34] and the monoacylglycerol lipase (MGLL) [35]. These genes commonly expressed in four tissues are involved in the top three significantly enriched strain- and tissue-specific canonical pathways NRF2-mediated oxidative stress response (head kidney/muscle, *p* = 2.37 × 10^−4^/4.97 × 10^−4^) and Glutathione biosynthesis (*p* = 2.10 × 10^−4^) in the Silver Steelhead strain and TREM1 signaling (*p* = 1.11 × 10^−4^) in the Born strain (Figure 2a).

### 3.2. TP53 Plays a Prominent Role as Upstream Regulator of Different Gene Expression Patterns in Both Trout Strains

The DE genes in the Silver Steelhead strain and the Born strain were assigned to a variety of canonical pathways using IPA. This included pathways of the stress and immune response (e.g., NRF2-mediated oxidative stress response, insulin-receptor signaling, TREM1 signaling, and Th1- and Th2-activation pathways), the cellular organization and development (ERK/MAPK signaling, protein-kinase-A signaling, ephrin-receptor signaling) as well as metabolic pathways (PPARα/RXRα activation and biotin-carboxyl-carrier-protein assembly). Among the top significantly enriched pathways shown in Figure 2a, five were affected uniquely in the Silver Steelhead strain when comparing the results for each tissue separately. These were IGF1 signaling (*p* = 1.3 × 10^−4^) and renin-angiotensin signaling (*p* = 2.42 × 10^−3^) in the head kidney, glutathione biosynthesis, and biotin-carboxyl-carrier-protein assembly (*p* = 2.10 × 10^−4^) in the liver, and PI3K/AKT signaling (*p* = 8.52 × 10^−3^) in the muscle tissue.

The comparison of the gene expression in both trout strains revealed a large number of differentially regulated genes but a distinct strain-specific pattern linked to a particular functional cluster was not evident. In this respect, only the regulation of pathways in the gills differed between the two rainbow trout strains might be commended. While in the Silver Steelhead strain, the top three predicted regulated pathways were assigned to the biofunctional categories “platelet activation”, “cell proliferation”, and “cytoskeletal organization”, the top activated pathways in the Born strain are involved in the innate and adaptive immune system, namely TREM1 signaling and pattern recognition receptors in recognition of bacteria and viruses, together with the T-cell exhaustion signaling pathway. 

In previous studies, we already observed considerable strain-specific differences in the branchial expression of innate immune genes in response to temperature [13] and infection [15]. This supports our assumption that the selective breeding of the Born strain in the challenging germ spectrum of brackish water is reflected by an adapted expression profile of immune genes in the gills, which are in direct contact with the environment.

We used the IPA tool to identify potential upstream regulators since that could have affected the altered gene expression [36] observed in the present study. Tumor protein P53 (*TP53*) appears to be the dominant upstream regulator in all tissues of the Silver Steelhead strain and in the head kidney, heart, and spleen of the Born strain (Figure 2b). Moreover, considerably more genes were regulated by *TP53* in several tissues (liver, gills, muscle, heart, spleen, and head kidney) of the Silver Steelhead strain than in the Born strain. Of note, the *TP53* gene is not differentially regulated between the two strains. P53 protein is one of the most intensively studied molecules to date [37,38]. It is considered the “guardian of the genome” [39] and the central stress responder in cells affecting a plethora of cellular processes such as cell cycle arrest, DNA repair, or the antioxidant response [38,40,41]. 

We found a transcriptional regulation of p53 target genes involved in a multitude of processes in the cells such as cycle progressing and growth (i.e., Silver Steelhead: *NDC80*, *CCNA2*, *CDK1*; Born: *CCNA2*, *EGFR*, *CSK*), metabolic processes (i.e., Silver Steelhead: *ACLY*, *COX10*, *ACAT1*; Born: *ABCA2*, *NDUFS1*, *ACADS*), stress and apoptotic pathways (i.e., Silver Steelhead: *SMURF2*, *DNAJA2*, *CASP6*, *BMF*; Born: *BNIP3*, *CASP8*, *CD82*, *GADD45A*), and the immune response (i.e., Silver Steelhead: *BCL6B*, *CXCR2*, *NFKBIA*; Born: *CASP1*, *IRF8*, *TNFSF9*).

### 3.3. Strain-Specific SNPs Were Identified in Silver Steelhead and Born Trout

The total number of putative SNPs detected from analysis 1 (A1) and analysis 2 (A2), both described in Section 2.4, when compared against the rainbow trout reference genome (GCA_002163495.1) were 675,619 and 211,541, respectively. The natural occurrence of gene mutations in the genome of a vertebrate is of an appreciable frequency. In the Yoruba population or European population of humans (*Homo sapiens*), 1.03 or 0.68 SNP, respectively, occur per kilobase [42]. The density of SNPs in the rainbow trout genome is significantly higher with one SNP every 64 base pairs (bp) [25]. This rate of polymorphism exceeds that of other teleosts such as channel catfish (*Ictalurus punctatus*: one SNP per 93 bp [43]), turbot (*Scophthalmus maximus*: one SNP per 302 bp [44]), and Atlantic salmon (*Salmo salar*: one SNP per 586 bp [45]). 

In this context, it should be noted that only some of these single nucleotide changes are polymorphic SNPs due to the additional salmonid-specific fourth round of whole-genome duplication (Ss4R) in salmonid fish [46,47]. Despite the natural process of rediploidization, the proportion of tetraploid genome regions still appears to be high [48]. Thus, the large number of remaining duplicated loci in rainbow trout can lead to a false identification of potential true polymorphic SNPs, because paralogous sequence variants (PSVs) and multi-site variants (MSVs) commonly occur in the genome of salmonid fish as well [49,50]. PSVs are non-polymorphic nucleotides that differ between paralogues/ohnologues genes aroused from a common ancestral sequence [51]. In contrast, MSVs are polymorphic and do segregate in one or both gene paralogues/ohnologues [50,52]. For example, Smith et al. (2005) assumed that up to one-third of the identified SNPs in Pacific salmon are paralogue sequence variants rather than true SNPs [44]. The number of true polymorphic SNPs identified in the present study is therefore most likely lower. However, besides the strain-specific gene-expression profiles, the two rainbow trout strains also differ in a variety of single nucleotide changes. 

After filtering the RNA-seq data for SNPs between both strains, we obtained information about the specific variants per tissue (A1) and over all tissues and individuals (A2). For A1 we detected a total of 37,000 putative SNPs, distributed over 7225 transcripts, 171 of which represented a shared polymorphism in the specific transcripts of all six tissues (Figure S1, Tables S4–S6). These results provided interesting information on shared polymorphisms throughout tissues, as well as, on SNPs that only affect transcripts of certain tissues. However, tissue-based analysis of SNPs differentiating both strains had the disadvantage of a reduced sample size, and with it a reduction of confidence in the genotype calls and coverage across genes. Thus, in the following we will focus only on the results of analysis 2. 

**Figure 3:**
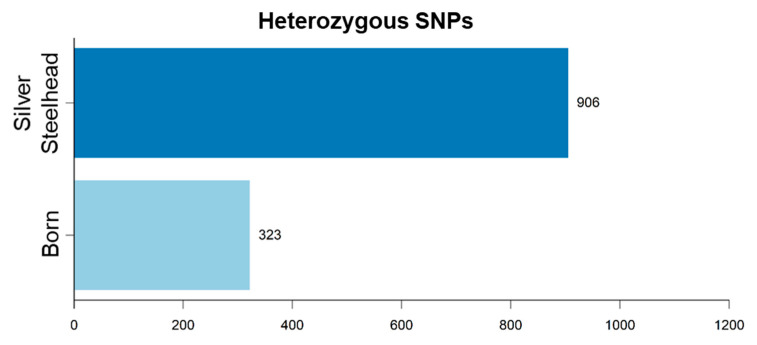
Number of heterozygous single nucleotide polymorphisms (SNPs) between the Silver Steelhead strain (dark blue) and the Born strain (light blue) over all tissues and individuals.

For A2, we detected a total of 1229 putative SNPs distinguishing both strains, from which 74% (906) were heterozygous for the strain Silver Steelhead and 26% (323) for the strain Born (Figure 3, Table S4). Nineteen of the putative SNPs were located in the DE genes *PRDX6*, *PSMA7*, *RPLP2*, *AHCY*, *DDOST*, *CEBPD*, *SLC6A13*, and *TSC22D1* (Table S3). Furthermore, 3686 and 16,630 of the 211,541 SNPs compared to the reference genome can be found on the rainbow trout 57K SNP [53] and 50K cSNP arrays, respectively [54]. 

We used the rainbow trout reference genome to assign and classify the 1229 putative SNPs across all tissues and individuals into three categories according to their genomic location and annotation. We also assigned the polymorphisms to their transcript-based regions: coding sequence (CDS), 3- and 5-prime untranslated region (UTR) (Table 2). 

The majority of the sequence variants were found within the CDS. Variations within the CDS are generally less frequent than those in non-coding regions due to the selection pressure [55]. Polymorphism can influence the functional conservation of the encoded gene product either directly, via non-synonymous mutations in the coding region, or indirectly, by variations in non-coding regions that change the transcriptional activity and/or translation efficiency [56,57]. The prevalence of SNPs inside the coding region in our study differs with the results verified for other teleost fishes such as Atlantic salmon [58] or Atlantic cod (*Gadus morhua*) [59].

**Figure 4:**
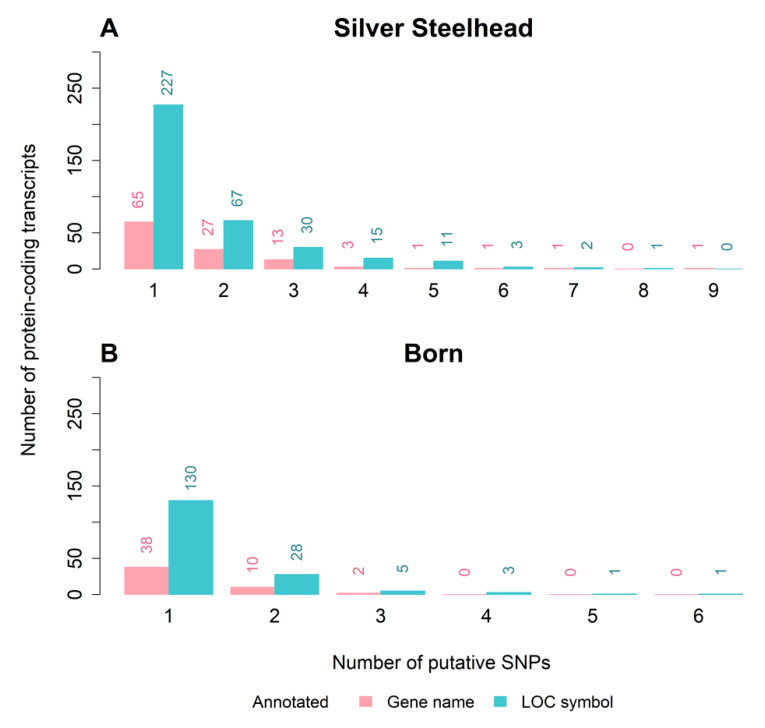
Number of heterozygous SNPs in annotated protein-coding transcripts with gene name (red) or LOC symbol (blue) in the Born strain (**A**) and the Silver Steelhead strain (**B**).

We subsequently classified putative SNPs belonging to the first category (exonic: with gene symbol) according to the length of the respective protein-coding transcripts (<1 kb, ≥1 to <3 kb, ≥3 to <5 kb, ≥5 kb, Table 3). Overall, most variants were assigned to transcripts with a length of ≥1 to <3 kb. Additionally, the distribution of the number of protein-coding transcripts by the number of putative heterozygous SNPs was determined for each strain (Figure 4). The majority of protein-coding transcripts contain only one SNP. This was consistent for both strains. Nevertheless, up to 9 or 6 SNPs in one gene could be identified in the Silver Steelhead strain (NM_001195009, heat shock protein 90 beta family member 1) and the Born strain (LOC110498860, predicted as histone H1.0-B-like), respectively. The SNP frequency per transcript corresponds to that found in sole (*Solea solea*) [60] or Japanese pufferfish (*Takifugu rubripes*) [61].

### 3.4. Putative SNPs Were Validated by Resequencing

**Figure 5:**
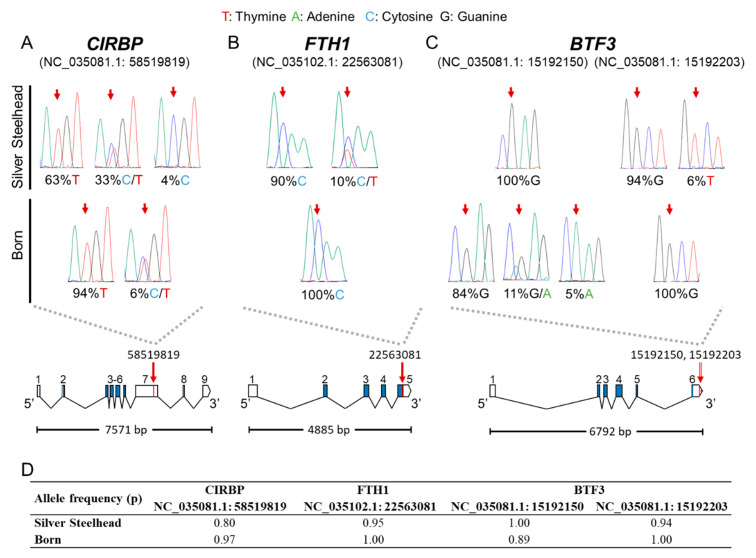
Validation and genomic location of four putative SNPs in rainbow trout strains Silver Steelhead and Born. Sequencing results are displayed by electropherograms (upper panel) showing the frequency of the sequence variants in the genes (**A**) *CIRBP* (NC_035081.1: 58519819), (**B**) *FTH1* (NC_035102.1: 22563081), (**C**) and *BTF3* (NC_035081.1: 15192150, NC_035081.1: 15192203). The lower panel displays the location of the SNPs (red dashes and arrows) in the respective gene structure. Exons are drawn by scaled boxes, and introns are shown as scaled lines. The coding region is highlighted in blue and the total transcript length is given under the gene structure. (**D**) Strain-specific allele frequency (p) for the validated SNPs.

We validated four of the putative SNPs by an approach combining PCR and capillary sequencing. The results confirmed the accuracy of the identified set of SNPs (Figure 5). The polymorphisms are located in the coding region or 3’UTR of the three genes *CIRBP* (NC_035081.1: rev/rs58519819; Figure 5A), *FTH1* (NC_035102.1: 22563081; Figure 5B), and *BTF3* (NC_035081.1, SNP1:15192150, SNP2:15192203; Figure 5C). While the non-synonymous SNPs in *CIRBP* and *BTF3* are located in their 3′-regions in exons 7 and 6, respectively, the synonymous SNP of *FTH1* resides in the coding region of exon 5. Synonymous SNPs do not directly lead to a change in the structure of the encoded protein and thus to putative changes in its functionality or effectiveness, but they can also have a significant impact [62]. It is commonly agreed that ongoing selection acting on genes involves both, synonymous and non-synonymous mutations [60,63,64]. 

### 3.5. Do the Identified Expression Differences and Genetic Variances Reflect a Specific Adaptation of the Born Strain?

Selective breeding can significantly improve the development and establishment of sustainable profitable aquaculture fish farming. Our study provides data on the genetic background of two rainbow trout breeding strains to cope with challenging conditions. The specific gene expression and SNP patterns support our assumption that the phenotype of the Born trout is adapted to regional conditions. Despite a relatively short anthropogenic selection period, this specific phenotype seems to be reflected in an altered genotype. On the other hand, it is known that aquaculture fish pass genetic bottlenecks during the periods of domestication and selection [65,66,67]. In the short term, this leads to certain improved traits such as growth rate or disease resistances. In the long run, a high risk of reduced performance might evoke as a consequence of a descending genetic quality and genetic diversity, which might culminate in inbreeding and genetic drift due to relatively small broodstocks [68,69]. Over time, this changes the gene frequency in the populations. Cossu et al. (2019) studied the influence of genetic drift on wild and farmed populations of the gilthead sea bream (*Sparus aurata*). They showed that genetic drift is one major driver shaping the genetic specificity of the farmed populations [70]. Taking this into account, the strong genetic variability of the Born strain is therefore most likely related to the interplay of adaptive selection and genetic drift. Association and simulation studies are necessary to determine the influence of each factor; however, they were not part of this study. The trait-specific association of the identified sequence variations and the influence of epigenetic patterns are potential subjects of future research to provide a better understanding of the observed genetic variations in terms of function.

## Figures and Tables

**Table 1:** Total number of sequencing reads obtained per strain and tissue.

Number of Reads		Raw	Trimmed
Total		345,567,818	323,864,488
Strains	Silver Steelhead	170,172,570	159,237,809
	Born	175,395,248	164,626,679
Tissue	Gills	47,055,697	44,162,122
	Head kidney	53,616,788	48,351,976
	Heart	52,003,354	49,537,095
	Liver	48,037,248	46,072,790
	Muscle	49,717,032	41,439,041
	Spleen	49,726,030	45,335,099

**Table 2:** Number of putative SNPs over all tissues and individuals according to heterozygosity, genomic regions and transcript regions.

Strain	Categories			Exonic Region		
	Exonic– Gene Symbol	Exonic– LOC Symbol	Others	CDS	5-Prime Region	3-Prime Region
**Silver Steelhead**	197	606	103	414	32	357
**Born**	64	224	35	160	15	113
**Total**	261	830	138	574	47	470

**Table 3:** Number of putative SNPs (exonic-gene symbol) over all tissues and individuals by length of protein-coding transcripts.

Length of Protein-Coding Transcripts	Over All Tissues and Individuals
<1 kb	62
≥1–<3 kb	165
≥3–<5 kb	23
≥5 kb	11
Total	261

## Supplementary Materials

The following are available online at https://www.mdpi.com/2073-4425/11/8/841/s1, Table S1: RIN values of RNA samples used for library preparation. Table S2: Primers used for SNP validation. Table S3: Lists of differentially expressed genes across all tissues and identified putative SNPs within differently expressed (DE) genes. Table S4: List of detected putative SNPs distinguishing the breeding strains. Table S5: Number of putative SNPs per tissue according to annotated genomic regions and per transcript region (A1). Table S6: Number of putative SNPs per tissue by length of protein-coding transcripts (A1). Figure S1: Venn diagram illustrating the identified SNPs that are common or exclusive to the six tissues.
