# Supplementary material for: Comparative Analysis of the Transcriptome and Distribution of Putative SNPs in Two Rainbow Trout (Oncorhynchus mykiss) Breeding Strains by Using Next-Generation Sequencing
Source: Genes (Basel). 2020 Jul 24;11(8):841. doi: 10.3390/genes11080841 (PMC7464081; doi:10.3390/genes11080841)
Supplement: Supplementary file 1 [file genes-11-00841-s001.zip › Table S5.docx]

| **Table S5.** Number of putative SNPs per tissue (based on analysis 1) according to annotated genomic regions and per transcript region. | | | | | | | | | |
| --- | --- | --- | --- | --- | --- | --- | --- | --- | --- |
| **Tissues** | **Categories** | | | |  | **Exonic Region** | | | |
|  | **Exonic** | | **Others** | **Total** |  | **CDS** | **5-prime region** | **3-prime region** | **Total** |
|  | **gene symbol** | **LOC symbol** |  |  |  |  |  |  |  |
| **Gills** | 1172 | 6131 | 1444 | 8747 |  | 2873 | 227 | 4203 | 7303 |
| **Head Kidney** | 1233 | 5830 | 1603 | 8666 |  | 2556 | 233 | 4274 | 7063 |
| **Heart** | 822 | 4073 | 1487 | 6382 |  | 1919 | 171 | 2805 | 4895 |
| **Liver** | 565 | 2193 | 822 | 3580 |  | 1377 | 115 | 1266 | 2758 |
| **Muscle** | 228 | 1288 | 311 | 1827 |  | 830 | 58 | 628 | 1516 |
| **Spleen** | 1045 | 5353 | 1400 | 7798 |  | 2670 | 253 | 3475 | 6398 |
