# Supplementary material for: Comparative Analysis of the Transcriptome and Distribution of Putative SNPs in Two Rainbow Trout (Oncorhynchus mykiss) Breeding Strains by Using Next-Generation Sequencing
Source: Genes (Basel). 2020 Jul 24;11(8):841. doi: 10.3390/genes11080841 (PMC7464081; doi:10.3390/genes11080841)
Supplement: Supplementary file 1 [file genes-11-00841-s001.zip › Table S6.docx]

| **Table S6.** Number of putative SNPs (exonic – gene symbol) per tissue (based on analysis 1) by length of protein-coding transcripts. | | | | | | |  |
| --- | --- | --- | --- | --- | --- | --- | --- |
| **Length of protein-coding transcripts** | **Tissue** | | | | | | |
|  | **Gills** | **Head Kidney** | **Heart** | **Liver** | **Muscle** | **Spleen** | |
| < 1 kb | 97 | 115 | 111 | 55 | 29 | 97 | |
| ≥ 1 - < 3 kb | 724 | 751 | 456 | 335 | 120 | 596 | |
| ≥ 3 - < 5 kb | 243 | 255 | 172 | 106 | 23 | 217 | |
| ≥ 5 kb | 108 | 112 | 83 | 36 | 7 | 135 | |
| **Total** | 1172 | 1233 | 822 | 532 | 179 | 1045 | |
| ^a^ A total of 33 (liver) and 49 (muscle) putative SNPs that could not be assigned to full length transcripts were excluded. | | | | | | |  |
