# Supplementary material for: Comparative Analysis of the Transcriptome and Distribution of Putative SNPs in Two Rainbow Trout (Oncorhynchus mykiss) Breeding Strains by Using Next-Generation Sequencing
Source: Genes (Basel). 2020 Jul 24;11(8):841. doi: 10.3390/genes11080841 (PMC7464081; doi:10.3390/genes11080841)
Supplement: Supplementary file 1 [file genes-11-00841-s001.zip › Figure S1_revised.PPTX]

## Slide 1
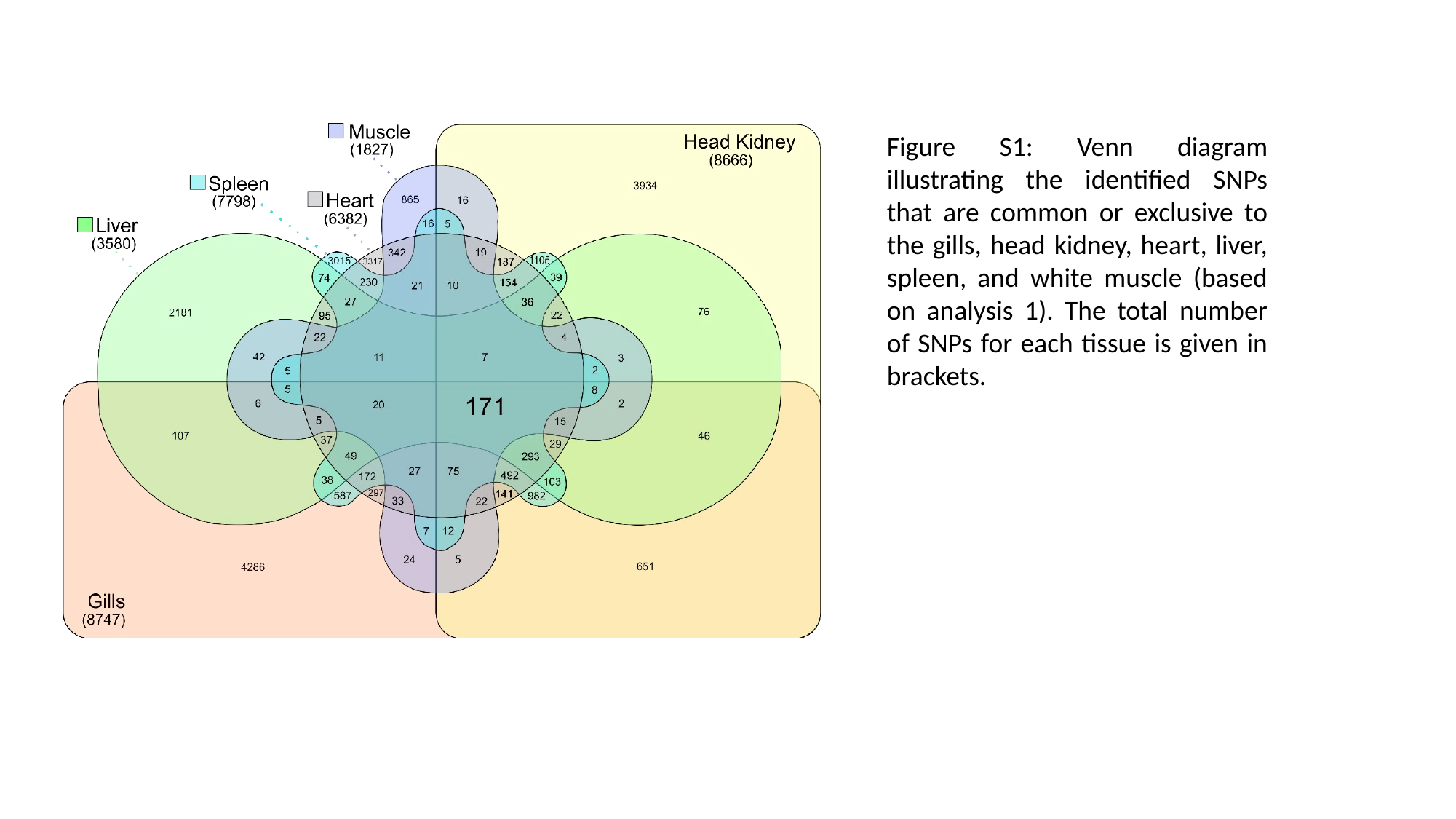

Figure S1: Venn diagram illustrating the identified SNPs that are common or exclusive to the gills, head kidney, heart, liver, spleen, and white muscle (based on analysis 1). The total number of SNPs for each tissue is given in brackets.
