# Supplementary material for: Comparative Analysis of the Transcriptome and Distribution of Putative SNPs in Two Rainbow Trout (Oncorhynchus mykiss) Breeding Strains by Using Next-Generation Sequencing
Source: Genes (Basel). 2020 Jul 24;11(8):841. doi: 10.3390/genes11080841 (PMC7464081; doi:10.3390/genes11080841)
Supplement: Supplementary file 1 [file genes-11-00841-s001.zip › Table S1.docx]

**Table S1.** RIN values of RNA samples used for library preparation

| **Sample** | **RIN value** | | | | | |
| --- | --- | --- | --- | --- | --- | --- |
|  | **Muscle** | **Heart** | **Head-Kidney** | **Spleen** | **Liver** | **Gills** |
| Born 1 | 9.8 | 9.9 | 9.5 | 9.4 | 10.0 | 9.7 |
| Born 2 | 9.5 | 9.7 | 9.6 | 9.6 | 9.6 | 9.7 |
| Born 3 | 9.6 | 9.8 | 10.0 | 9.6 | 10.0 | 9.5 |
| Born 4 | 9.5 | 10.0 | 10.0 | 10.0 | 10.0 | 10.0 |
| Born 5 | 10.0 | 9.9 | 10.0 | 10.0 | 10.0 | 9.5 |
| Born 6 | 9.9 | 9.9 | 10.0 | 10.0 | 8.5 | 10.0 |
| Born 7 | 9.3 | 10.0 | 10.0 | 9.9 | 10.0 | 10.0 |
| Born 8 | 9.9 | 9.9 | 10.0 | 10.0 | 10.0 | 9.9 |
| Silver Steelhead 1 | 9.2 | 9.0 | 8.7 | 9.2 | 9.7 | 9.7 |
| Silver Steelhead 2 | 8.8 | 9.3 | 10.0 | 9.2 | 9.9 | 9.1 |
| Silver Steelhead 3 | 9.3 | 9.4 | 9.9 | 9.4 | 9.6 | 8.9 |
| Silver Steelhead 4 | 9.8 | 10.0 | 9.3 | 10.0 | 9.9 | 9.7 |
| Silver Steelhead 5 | 9.7 | 9.7 | 9.7 | 9.8 | 10.0 | 8.6 |
| Silver Steelhead 6 | 9.5 | 9.9 | 10.0 | 10.0 | 10.0 | 9.8 |
| Silver Steelhead 7 | 9.8 | 9.8 | 9.2 | 10.0 | 9.8 | 9.0 |
| Silver Steelhead 8 | 9.7 | 9.7 | 9.5 | 10.0 | 10.0 | 9.8 |
