# Supplementary material for: Comparative Analysis of the Transcriptome and Distribution of Putative SNPs in Two Rainbow Trout (Oncorhynchus mykiss) Breeding Strains by Using Next-Generation Sequencing
Source: Genes (Basel). 2020 Jul 24;11(8):841. doi: 10.3390/genes11080841 (PMC7464081; doi:10.3390/genes11080841)
Supplement: Supplementary file 1 [file genes-11-00841-s001.zip › Table S2.docx]

**Table S2 Primers used for SNP validation**

| **Primer name** | **Sequence 5’-3’** | **GenBank accession** |
| --- | --- | --- |
| BTF3_f1 | TCTTGCCACCCGATTCTCCAAA | NC_035081.1: 15185498-15192249 |
| BTF3 _r1 | GTTTATTCCAGATATTATGGTTTCAG |  |
| CIRBP_f1 | TTACTTTGACTGGTGAACCTACTT | NC_035081.1: 58517314-58524885 |
| CIRBP_r1 | TAATATGCTGATGGACTGAACGTT |  |
| FTH1_f1 | AGTTGGGTGACTGGGTGACCA | NC_035102.1: 22558458-22563342 |
| FTH1_r1 | ATTCGCGCTCCTTCCCCAAAG |  |
